# Perinatal care and its association with perinatal death among women attending care in three district hospitals of western Uganda

**DOI:** 10.1186/s12884-024-06305-5

**Published:** 2024-02-06

**Authors:** Mercy Muwema, Joaniter I. Nankabirwa, Grace Edwards, Gorrette Nalwadda, Joanita Nangendo, Jaffer Okiring, Gloria Odei Obeng-Amoako, Wilson Mwanja, Elizabeth N. Ekong, Joan N. Kalyango, Dan K. Kaye

**Affiliations:** 1 Clinical Epidemiology Unit, School of Medicine, College of Health Sciences, Makerere University, Kampala, Uganda; 2 Infectious Diseases Research Collaboration, Kampala, Uganda; 3 School of Nursing and Midwifery, Aga Khan University, Kampala, Uganda; 4 Department of Nursing, School of Health Sciences, College of Health Sciences, Makerere University, Kampala, Uganda; 5 Department of Nutrition and Food Science, School of Biological Sciences, College of Basic and Applied Sciences, University of Ghana, Legon, Ghana; 6 Whale Consult Limited, Kampala, Uganda; 7 Department of Nursing and Midwifery, Faculty of Public Health, Nursing and Midwifery, Uganda Christian University, Mukono, Uganda; 8 Department of Pharmacy, School of Health Sciences, College of Health Sciences, Makerere University, Kampala, Uganda; 9 Department of Obstetrics and Gynecology, School of Medicine, College of Health Sciences, Makerere University, Kampala, Uganda

**Keywords:** Antenatal care, Intrapartum care, Postnatal care, Stillbirth, Neonatal death, Perinatal death, Birth outcomes

## Abstract

**Background:**

Provision of effective care to all women and newborns during the perinatal period is a viable strategy for achieving the Sustainable Development Goal 3 targets on reducing maternal and neonatal mortality. This study examined perinatal care (antenatal, intrapartum, postpartum) and its association with perinatal deaths at three district hospitals in Bunyoro region, Uganda.

**Methods:**

A cross-sectional study was conducted in which a questionnaire was administered consecutively to 872 postpartum women before discharge who had attended antenatal care and given birth in the study hospitals. Data on care received during antenatal, labour, delivery, and postpartum period, and perinatal outcome were extracted from medical records of the enrolled postnatal women using a pre-tested structured tool. The care received from antenatal to 24 h postpartum period was assessed against the standard protocol of care established by World Health Organization (WHO). Poisson regression was used to assess the association between care received and perinatal death.

**Results:**

The mean age of the women was 25 years (standard deviation [SD] 5.95). Few women had their blood tested for hemoglobin levels, HIV, and Syphilis (*n* = 53, 6.1%); had their urine tested for glucose and proteins (*n* = 27, 3.1%); undertook an ultrasound scan (*n* = 262, 30%); and had their maternal status assessed (*n* = 122, 14%) during antenatal care as well as had their uterus assessed for contraction and bleeding during postpartum care (*n* = 63, 7.2%). There were 19 perinatal deaths, giving a perinatal mortality rate of 22/1,000 births (95% Confidence interval [CI] 8.1–35.5). Of these 9 (47.4%) were stillbirths while the remaining 10 (52.6%) were early neonatal deaths. In the antenatal phase, only fetal examination was significantly associated with perinatal death (adjusted prevalence ratio [aPR] = 0.22, 95% CI 0.1–0.6). No significant association was found between perinatal deaths and care during labour, delivery, and the early postpartum period.

**Conclusion:**

Women did not receive all the required perinatal care during the perinatal period. Perinatal mortality rate in Bunyoro region remains high, although it’s lower than the national average. The study shows a reduction in the proportion of perinatal deaths for pregnancies where the mother received fetal monitoring. Strategies focused on strengthened fetal status monitoring such as fetal movement counting methods and fetal heart rate monitoring devices during pregnancy need to be devised to reduce the incidence of perinatal deaths. Findings from the study provide valuable information that would support the strengthening of perinatal care services for improved perinatal outcomes.

**Supplementary Information:**

The online version contains supplementary material available at 10.1186/s12884-024-06305-5.

## Introduction

For countries to meet the national Sustainable Development Goal 3 targets to: (1) reduce the maternal mortality ratio to 70 deaths/100,000 live births by 2030 [1]; (2) reduce neonatal mortality rate to 12/1000 live births by 2030; and (3) reduce still births to 10 or less stillbirths/1000 total births as indicated in the WHO Every Newborn Action plan [2], there is need to improve the quality of perinatal care [3]. Indeed, provision of effective care to all women and newborns during delivery in health facilities has been estimated to prevent approximately 113,000 maternal deaths, 531,000 still births, and 1.325 million neonatal deaths annually [4].

Quality of care is defined by World Health Organization (WHO) as the extent to which health services given to individuals improve their desired health outcomes. For this to happen, the healthcare needs to be safe, effective, timely, efficient, equitable and people-centred [5]. To improve the quality of care during the perinatal period, WHO proposes a number of health care recommendations, including their time, frequency and method of provision to be given to the women during the antenatal, labour and delivery, and postnatal periods [6–8]. The WHO recommendations were designed to diagnose, prevent, and treat causes of maternal and neonatal deaths, and stillbirths [5]. As part of these recommendations, mothers are encouraged to have at least four antenatal care visits, have their labour monitored using a partograph, and receive postnatal care for the first 24 h. However, even when the number of women receiving this care tends to be generally satisfactory [9–12], neonatal mortality and stillbirth rates have remained high in Sub-Saharan Africa [13, 14].

Studies conducted to assess the associations between improved perinatal care and birth outcomes have had positive conclusions. A study that assessed the impact of implementing the 2016 WHO recommendations on antenatal care in South Africa revealed a 5.8% absolute decrease in stillbirths with the implementation of the recommendations [15]. A study conducted in Ethiopia showed that complete providers’ adherence to the recommendations during antenatal care was significantly associated with a decrease in neonatal complications [16]. Finally, a study in Uganda showed that having four or more antenatal visits reduced the odds of stillbirth, early neonatal death, low birth weight, and a 5-minute Apgar score of less than seven [17]. For the intrapartum period, studies have shown that the use of a partograph to monitor the indicated parameters during labour reduces birth asphyxia, a cause of perinatal deaths [18].

Although the studies above have shown a positive impact of adopting the WHO recommendations on birth outcomes, they have been limited to independent phases of care and not the full spectrum of care including the antenatal, intrapartum and postnatal care. In this study we document the association between perinatal care received during the antenatal, intrapartum, and postnatal periods, and perinatal deaths.

## Methods

### Study design and setting

A cross-sectional study was conducted between March and June 2020 in the three public district hospitals of Bunyoro region, Uganda. The region is comprised of eight districts that include Kakumiro, Kibaale, Kagadi, Kikuube, Hoima, Masindi, Buliisa, and Kiryandongo [19]. The region’s population was estimated at 2,028,500 million people in the 2014 National Demographic and Population Census [20]. Bunyoro region has one of the highest fertility rates in the country (7.5 births per woman), has a high proportion of teenage pregnancies (10.6%), and a high prevalence of early marriages (19%) [21]. The region has three district hospitals (Kagadi, Kiryandongo and Masindi). A district hospital is the highest-level public health facility in any given district and covers a catchment population of approximately 500,000 people. The district hospitals offer preventive, promotive, and both in and out patient curative services in all areas of child and adult medicine [22]. The hospitals are also responsible for supervising and planning for all the lower-level facilities within the district. An average of 300 births and eight [8] perinatal deaths are registered at each of these hospitals in any given month.

### Study population

Women in their early postpartum period receiving care in the three participating district hospitals were screened at discharge for eligibility to join the study. A mother was eligible for inclusion in the study if: (1) she attended antenatal care in the study hospitals; (2) she gave birth in the study hospitals; (3) birth was conducted by a skilled health professional; (4) she provided written informed consent to participate in the study; and (5) she had a health record indicating care received during the antenatal, intrapartum, and postpartum periods.

### Sample size and sampling

We hypothesized that occurrence of perinatal death would be different between women receiving the recommended perinatal care and those not receiving the care recommended. Using sample size formula for comparison of two proportions, and estimates of the prevalence of perinatal death among women who received the recommended care to be 2.5% and 5.3% among those who had not received based on a study done in Uganda [17], a sample size of 825 would be sufficient to test the hypothesis; assuming 5% level of significance, 80% power, adjusting for a design effect of 2 to cater for clustering at health facility level, and a non-response of 10%. We determined the number of women to be enrolled from each hospital using probability proportionate to size based on postnatal data from the hospitals. One hundred and ninety (190), 317, and 318 women were to be selected from Kiryandongo, Kagadi, and Masindi hospitals respectively. Consecutive sampling within each of the hospitals was used to select participants for the study.

### Data collection

At each hospital, data was collected by qualified midwives fluent in the local language of the area (Runyoro) and not directly involved in the routine patient care at the participating hospitals. Women in their early postpartum period were identified using the ward registers and screened for eligibility to join the study. Following the consent process, a questionnaire was administered to collect socio-demographic data from the participants. Data on care received from start of antenatal care to 24 h postpartum and perinatal outcome was extracted from the participants’ hospital notes using a pre-tested structured data extraction tool designed using the Open Data Kit (ODK) software.

The dependent variable in this study was either a perinatal death or a live baby. Perinatal death referred to a documented still birth (death after 28 weeks of pregnancy before or during birth) or a neonatal death (death in the first 24 h after birth). This data was extracted from the women’s discharge forms or patient files. The independent variables included: antenatal care, intrapartum care, and postpartum care within 24 h after birth.

Antenatal care comprised of: number of antenatal contacts, time of initiation of antenatal care, examinations (fetal status and maternal status) during every contact, urine and blood tests conducted, ultrasound scan, prophylaxis for anaemia and malaria, and immunization against tetanus. This data was extracted from the women’s antenatal cards or mother child health passports. Intrapartum care included: fetal heart rate monitoring (fetal condition), progress of labour monitoring (cervical dilatation, descent of presenting part, and uterine contractions), and maternal condition monitoring (blood pressure and pulse rate measurements). Data on intrapartum care was extracted from the women’s partographs or patient files. Finally, the postpartum care within 24 h after birth considered by the study included: uterine assessments, maternal vital status monitoring, and urine voiding assessment. In particular, uterine assessment involved examination for uterine contraction, vaginal bleeding, and fundal height, while maternal vital status assessment included measuring of the woman’s blood pressure, temperature, and pulse rate. Postpartum care data was extracted from the women’s patient files.

### Data management and statistical analysis

Data collected were exported to STATA version 13 for cleaning and analysis. For perinatal death (primary outcome), a score of 1 was assigned if a woman had a perinatal death and a score of 0 if a woman did not have a perinatal death. Each care parameter received by women during the perinatal period was assigned a score of 1 if it was received at least once during the entire period while a score of 0 if it was not received at all during the entire period. Antenatal contacts were categorized into less than four and four or more contacts as used in previous studies of pregnancy outcome to allow generalizability and comparison of results, although the current guidelines recommend eight contacts to be sufficient contact [17, 23, 24].

Continuous variables (age, income and parity) were categorized at analysis and all variables were summarized and presented as proportions stratified by the perinatal outcome. Perinatal mortality rate, stillbirth rate, and early neonatal death rate were calculated based on the WHO definition. Perinatal mortality was defined as the number of perinatal deaths per 1000 births; stillbirth rate as the number of stillbirths per 1000 total births; and the early neonatal death rate as the probability that a child born alive died during the first 24 h after birth, expressed per 1000 live births [25]. The mortality rates were then adjusted for clustering.

Modified Poisson with robust standard errors was used to assess for the association between perinatal deaths and perinatal care. Separate models were built for each level of care including the antenatal, intrapartum and postpartum care. All variables that had *p*-value of less than or equal to 0.20 at bivariate analysis were considered for multivariate analysis and logical model building was used to generate the final models. In the antepartum model, education level and ANC prophylaxis were found as confounders while maternal status monitoring, ultrasound scan, health facility attended, blood tests, ANC start trimester, and ANC contacts were considered as known confounders. All eight were included in the final model even when not statistically significant. For the intrapartum model, education level was a confounder while fetal condition assessment and labour progress assessment are known confounders, so all were included in the final model. Finally postpartum model was confounded by the education level and health facility attended while uterine assessment was considered a known confounder and all three were included in the final model. For all models, the measures of associations are presented as prevalence ratio (PR) with their 95% confidence intervals and *p*-values. A *p*-value of < 0.05 is considered significant.

### Ethical considerations

Ethical approval to conduct the study was obtained from the Makerere University School of Medicine Research and Ethics Committee (REC REF# 2019 − 137) and the Uganda National Council for Science and Technology (HS483ES). Written informed consent to participate in the study was obtained from all participants prior to enrolment in the study, and unique identifiers and not personal names were used for participant identification.

## Results

### Description of the study population

**Fig. 1:**
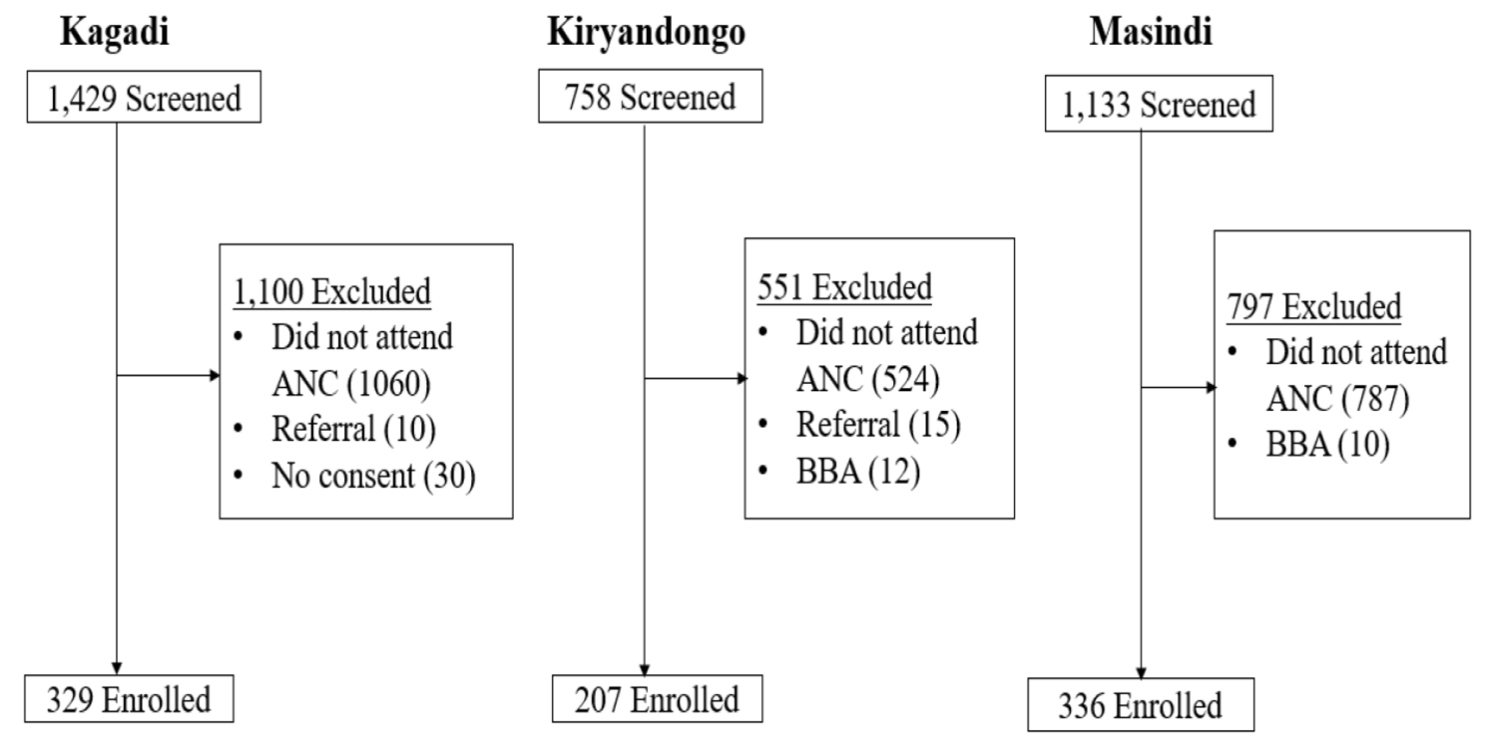
Flow chart

A total of 3,320 women were screened for eligibility to join the study, and 872 (26.3%) were enrolled. The commonest reason for exclusion was having not attended antenatal care at the study hospitals where they gave birth from (2,371, 96.9%). Other reasons for exclusion included patients being referred to study hospitals from lower facilities due to intrapartum complications, mothers arriving at the facility after birth (birth before arrival (BBA)) and refusing to provide informed consent to participate in the study. Figure 1 provides details of the participants flow stratified by health facility.

The mean age (standard deviation, SD) of the 872 enrolled participants was 25 (5.95) years. Majority of the participants were married or in a stable relationship (*n* = 782, 89.7%), and more than half (*n* = 453, 52%) had never received any formal education. Although many of the participants had two or more children (*n* = 615, 70.5%), almost all (*n* = 846, 97%) earned less than 500,000 Uganda Shillings ($140) per month. Table 1 provides details of the characteristics of the study participants.

### Perinatal deaths among the study participants

Of the 872 women enrolled in the study, 9 (1%) had still birth giving a stillbirth rate of 10 deaths per 1000 total births (95% CI -1.9–22.5). In addition, 10 (1.2%) women lost their babies within the first 24 h of their birth giving the early neonatal death rate of 12 deaths per 1000 live births (95% CI -5.5–28.4). Overall, 19 women lost their babies in the perinatal period resulting in a perinatal mortality rate of 22 deaths per 1000 births (95% CI 8.1–35.5). Among the women who had perinatal death, 10 (52.6%) had delivered by spontaneous vaginal delivery and 9 (47.4%) were delivered by a C-section. No perinatal death was observed among women with assisted vaginal delivery. Most of the deaths were among women who earned less than UGX 100,000 ($28.6) per month compared to those who earned a higher salary (15, 79.0% versus 4, 21.0% respectively, *p* < 0.001). Similarly, most of the perinatal death were observed in women with no/only primary education, aged 20 to 35 years of age, married, and with no employment (Table 1). Perinatal deaths were mainly caused by childbirth related complications (15/19, 78.9%) specifically birth asphyxia [14] and fetal head retention [1]. The other causes of death were prolonged pregnancy [1], birth defects [1], suffocation [1], and unknown cause [1]. Birth asphyxia resulted from prolonged, obstructed labour, and prolapsed cord.

**Table 1:** Characteristics of the study population stratified by outcomes

Characteristic	Live birth *N* = 853 *n* (%)	Perinatal death *N* = 19 *n* (%)	Total *N* = 872 *n* (%)	*p*-value
Facility				0.63
Kagadi	322(37.8)	7(36.8)	329(37.7)	
Kiryandongo	204(23.9)	3(15.8)	207(23.7)	
Masindi	327(38.3)	9(47.4)	336(38.5)	
Mode of delivery				< **0.001**
Spontaneous vaginal delivery	720(84.4)	10(52.6)	730(83.7)	
Assisted vaginal delivery	11(1.3)	0(0)	11(1.3)	
Caesarian Section	122(14.3)	9(47.4)	131(15.0)	
History of admission during pregnancy				0.98
No	586(68.7)	13(68.4)	599(68.7)	
Yes	267(31.3)	6(31.6)	273(31.3)	
Age of the mother				0.78
< 20	162(19.0)	3(15.8)	165(18.9)	
20–35	634(74.3)	14(73.7)	648(74.3)	
>35	57(6.7)	2(10.5)	59(6.8)	
Education level				0.56
None/Primary	441(51.7)	12(63.2)	453(52.0)	
Secondary	320(37.5)	6(31.6)	326(37.4)	
Tertiary	92(10.8)	1(5.3)	93(10.7)	
Marital status				< **0.001**
Married/Stable relationship	769(90.2)	13(68.4)	782(89.7)	
Single/divorced/separated	84(9.8)	6(31.6)	90(10.3)	
Occupation				0.23
None	288(33.8)	10(52.6)	298(34.2)	
Informal employment	486(57.0)	8(42.1)	494(56.6)	
Formal employment	79(9.3)	1(5.3)	80(9.2)	
Income				**0.02**
<100,000/=	405(47.5)	15(79.0)	420(48.2)	
100,000–500,000/=	422(49.5)	4(21.0)	426(48.8)	
>500,000/=	26(3.1)	0(0.0)	26(3.0)	
Parity				0.40
Primigravida (1)	249(29.2)	8(42.1)	257(29.5)	
Low parity (2–3)	326(38.2)	7(36.8)	333(38.2)	
Multipara (> 3)	278(32.6)	4(21.1)	282(32.3)	

### Antenatal care components associated with perinatal death

In the antenatal period, most women received prophylaxis for anaemia, malaria, and tetanus, and had their fetal status monitored at least once during the entire period (*n* = 741, 85% and *n* = 852, 97.7% respectively). However, a few women had their hemoglobin levels estimated nor their blood tested for HIV and syphilis (*n* = 53, 6.1%); had their urine tested for glucose and proteins (*n* = 27, 3.1%); took an ultrasound scan (*n* = 262, 30%); and had their BP, weight, and pallor (maternal status) assessed (*n* = 122, 14%). No perinatal death was observed among women who had eight or more contacts and had their urine tested for glucose and proteins. Details of the antenatal care received by women are provided in Supplement Table 1.

The prevalence of perinatal death was significantly lower among women whose fetal status examination (Fetal Heart Rate (FHR), Lie, Position, Fundal Height (FH)) was conducted during antenatal contacts compared to those who did not have it done (aPR = 0.22, 95% CI 0.1–0.6). The prevalence of perinatal death was also lower among women who attended antenatal care four or more times (aPR = 0.87, 95% Cl 0.3–2.3), and those who had their blood tested for hemoglobin level, HIV and syphilis (aPR = 0.88, 95% CI 0.1–6.8), although these associations were not statistically significant. However, the reverse was noted among women who undertook an ultrasound scan (aPR = 1.72, 95% Cl 0.6–4.8); had their blood pressure, weight and pallor assessed (aPR = 1.72, 95% Cl 0.6–4.6); and received iron/folic acid, fansidar, and tetanus toxoid during pregnancy (aPR = 1.96, 95% Cl 0.5–8.3). These women experienced a higher prevalence of perinatal death despite the associations not being statistically significant compared to those who did not undertake these interventions. Details of the associations between antenatal care received and perinatal death are provided in Table 2.

**Table 2:** Association of ANC care components with perinatal death

Characteristic	*n/N* (%)	Unadjusted PR (95% CI)	*P*-value	Adjusted PR (95% CI)	*P*-value
Number of ANC contacts					
< 4 contacts	9/406(2.2)	1.00		1.00	
4 or more contacts	10/466(2.2)	0.97(0.4–2.4)	0.94	0.87(0.3–2.3)	0.78
Initiation of ANC care					
1st trimester	3/73(4.1)	1.00		1.00	
After 1st trimester	16/799(2.0)	0.49(0.2–1.6)	0.25	0.67(0.2–2.1)	0.50
Prophylaxis (Fe/Fo, SP, TT)					
No	2/131(1.5)	1.00		1.00	
Yes	17/741(2.3)	1.50(0.4–6.4)	0.58	1.96(0.5–8.3)	0.36
Blood testing (HIV, Syphilis, Hb)					
No	18/819(2.2)	1.00		1.00	
Yes	1/53(1.9)	0.86(0.1–6.3)	0.88	0.88(0.1–6.8)	0.90
Ultrasound scan					
No	12/610(2.0)	1.00		1.00	
Yes	7/262(2.7)	1.36(0.5–3.4)	0.52	1.72(0.6–4.8)	0.30
Fetal status examination (FHR, Lie, Position, FH)					
No	2/20(10.0)	1.00		1.00	
Yes	17/852(2.0)	0.20(0.1–0.8)	0.02	0.22(0.1–0.6)	**0.01**
Maternal status examination (BP, Wt, Pallor)					
No	14/750(1.9)	1.00		1.00	
Yes	5/122(4.1)	2.20(0.8–6.4)	0.13	1.72(0.6–4.6)	0.28

*Fe/Fo: Ferrous sulphate Folic acid

SP: Fansidar

TT: Tetanus Toxoid

FHR: Fetal Heart Rate

FH: Fundal Height

BP: Blood Pressure

Wt: Weight

### Labour, delivery, and first 24 h maternal postpartum care components associated with perinatal death

During labour and delivery, most women had their labour monitored at least once for fetal condition (fetal heart rate) (*n* = 660, 75.7%), maternal condition (blood pressure and pulse rate) (*n* = 505, 57.9%), and labour progress (cervical dilatation, descent of presenting part, and uterine contraction) (*n* = 611, 70.1%). Details of the care received by women are provided in Supplement Table 1. The prevalence of perinatal death was lower among women whose fetal condition was assessed during labor (aPR = 0.88, 95% Cl 0.0-21.2), maternal condition was measured including blood pressure and pulse rate (aPR = 0.29, 95% CI 0.1–1.1), and whose progress of labour was monitored including cervical dilatation, descent of presenting part, uterine contraction (aPR = 0.94, 95% CI 0.1–19.5), although these associations were not statistically significant (Table 3).

Lastly, in the first 24 h of the postpartum period, most women had their BP, pulse rate and temperature (maternal status) taken at least once (*n* = 533, 61.1%). However, only 7.2% (*n* = 63) of the women had their uterus assessed for contraction and bleeding (uterine assessment) (Supplement Table 1). The prevalence of perinatal death was higher among women whose uterus (uterine contraction, vaginal bleeding, FH) and maternal status (BP, PR, temperature) were assessed 24 h after birth compared to those who were not assessed although the associations were not statistically significant (aPR = 2.40, 95% Cl 0.8–6.9 for women whose uterine assessment was done and aPR = 1.41, 95% CI 0.3–6.7 for women whose maternal status was assessed) (Table 3).

**Table 3:** Association of intrapartum and postpartum care components with perinatal death

Characteristic	*n/N* (%)	Unadjusted PR (95% CI)	*P*-value	Adjusted PR (95% CI)	*P*-value
**Intrapartum**					
Fetal condition assessment					
No	8/212(3.8)	1.00		1.00	
Yes	11/660(1.7)	0.44(0.2–1.1)	0.07	0.88(0.0-21.2)	0.94
Maternal condition assessment					
No	13/367(3.5)	1.00		1.00	
Yes	6/505(1.2)	0.34(0.1–0.9)	0.03	0.29(0.1–1.1)	0.06
Labour progress assessment					
No	9/261(3.5)	1.00		1.00	
Yes	10/611(1.6)	0.47(0.2–1.2)	0.10	0.94(0.1–19.5)	0.97
**Postpartum**					
Uterine assessment (uterine contraction, vaginal bleeding, FH)					
No	15/809(1.9)	1.00		1.00	
Yes	4/63(6.4)	3.42(1.2–10.0)	0.03	2.40(0.8–6.9)	0.11
Maternal status assessment (BP, PR, Temperature)					
No	7/339(2.1)	1.00		1.00	
Yes	12/533(2.3)	1.09(0.4–2.7)	0.85	1.41(0.3–6.7)	0.66

## Discussion

This study found a perinatal mortality rate of 22 per 1,000 births, comprised by a stillbirth rate of 10 per 1,000 total births and early neonatal mortality rate of 12 per 1,000 live births. Most perinatal deaths occurred among women who delivered spontaneously per vagina; had no/or only primary education; earned less than 100,000UGX ($28.6) per month; and were married. Prevalence of perinatal death was lower among women whose fetus was monitored during antenatal; who attended antenatal care four or more times; had their blood tested for HIV, syphilis, and hemoglobin level; and had their labour and delivery monitored for the fetal state, maternal state, and labour progress.

The estimated perinatal mortality in this setting is quite high with at least 2 in every 100 women having a perinatal death. This high number of perinatal deaths indicates that quality of care received during the perinatal period may have a direct impact on perinatal outcomes. However, although the perinatal mortality is high, it is still lower than the rates observed in earlier studies in Uganda that is 58(3%) stillbirths and 198 (11%) poor birth outcome in Western Uganda, 1432 (4.2%) late stillbirths and 495 (1.8%) early neonatal death in Eastern Uganda, and perinatal mortality of 43/1000 births in Northern Uganda [17, 26, 27] and also lower than the estimated perinatal mortality rates for the same region (institutional perinatal mortality of 26.1/1000 births) [28]. This difference in estimated mortality could be attributed to the stringent inclusion criterion used for women to join this study. In order to reduce on the missing data from the records, we excluded women who were emergency referrals, and those who did not attend antenatal care or delivered outside the study hospitals. Unfortunately, risk of perinatal mortality tends to be higher in women who are referred and those that do not attend antenatal care [29, 30], meaning that the actual mortality rates may be higher than those estimated by the study.

Perinatal mortality was common among women who had spontaneous vaginal deliveries compared to other forms of deliveries. Even with the supervision of a trained health worker, vaginal deliveries may have higher risks of perinatal deaths than operative deliveries. This could be because of malpresentations that may not be easily corrected due to limited time for manipulation, emergency deliveries when there are pregnancy complications like pre-eclampsia and eclampsia, multiple gestation, and preterm deliveries resulting in adverse outcomes [31, 32]. This observation is similar to findings from other studies including a study conducted in Ghana which showed a 5.9% prevalence of stillbirths among women who had delivered vaginally [31].

The higher prevalence of perinatal deaths observed among married women than the single women may be a unique finding to this study. Previous studies have shown that single/separated/divorced women are likely to have higher risk of perinatal death than married women due to the social, mental and monitory support they receive from their spouses [33, 34]. The difference observed in this study could be a result of the wealth status of the women in this particular region despite being married. Women in Bunyoro may not be fully empowered and supported by their spouses to seek services during pregnancy and childbirth, and thus the benefits of being married may not be appreciated in this setting [35].

Women earning less than UGX 100,000 ($28.6) per month were also observed to have more perinatal deaths than those earning more. This finding confirms results from earlier studies that have shown a higher risk of perinatal death among poor women in developing countries [34, 36, 37]. This could explain the occurrence of perinatal death among women who had less than eight contacts and had not undertaken an ultrasound scan in this study. Women who earn less are less likely to effectively use maternal health services like antenatal care which are known to reduce the risk of perinatal death even when they are free of charge due to their inability to meet the indirect costs like transport and personal needs [38]. Strategies aiming at economic empowerment of women could improve their access to maternal health services.

Similarly, women whose pregnancies were assessed at least once for fetal heart rate, fetal lie, and position, and fundal height during the antenatal period were observed to significantly have a lower prevalence of perinatal death. This confirms the protective nature of the fetal status monitoring intervention which identifies perinatal death risk factors like fetal distress for better management [39]. A lower prevalence was also seen among women whose blood was tested for HIV, syphilis and hemoglobin; attended antenatal four or more times; and started antenatal care late though the relationship was not significant. This emphasizes the importance of maternal screening in identification and management of perinatal death risk factors [39–41]. Therefore, availability of equipment, diagnostic tests, and adequate numbers of skilled health care providers could increase on the frequency and number of women assessed coupled with proper management of the identified risks. Lack of these characterizes resource constrained settings like Uganda [42–44].

Our findings however differ from previous studies in regard to antenatal contacts and time of start for antenatal care. Previous studies found increased antenatal contacts and early starting of antenatal care to significantly reduce the occurrence of perinatal death [17, 45, 46]. The difference in findings observed in this study could be a result of the low proportions of early initiation of ANC, ANC attendance of 4 and above, and stillbirth, and exclusion of referrals in this study; indulgence in self-care among experienced women [47]; and the fact that health care providers perceive women who start ANC late to be at risk and thus maximize care to them.

Despite the relationship not being statistically significant, generally women whose labour was monitored to assess fetal heart rate (fetal condition), maternal blood pressure and pulse rate (maternal condition), and cervical dilatation, descent of presenting part, and uterine contraction (labour progress) were seen to have fewer perinatal deaths. These intrapartum interventions when used are known to save life [39, 48]. These findings are in agreement with findings of a study conducted in Ethiopia, Zanzibar and Nepal respectively where women whose fetal heart rate, cervical dilatation, uterine contractions, and blood pressure were not monitored as recommended had higher odds of experiencing stillbirths [48–50]. This further calls for well-trained health care providers, sufficient infrastructure, and availability of necessary equipment.

Women assessed for uterine and maternal status immediately after birth experienced more perinatal deaths compared to those who were not. The assessment of these women by health care providers during the postnatal period could have been influenced by their health state during antenatal or labour and delivery. Women often experience pregnancy related complications or undergo operative delivery that could be associated with perinatal death [51, 52]. This calls for closer monitoring and observation by the health care providers during the postnatal period. Women who have experienced complications or undergone operative delivery are at risk of experiencing perinatal deaths due to an already compromised state of the baby [18] and compromised breastfeeding resulting from mother’s poor health state [53]. However, this finding contrast an earlier study conducted in five countries of Bangladesh, Ethiopia, Ghana, Guinea-Bissau and Uganda which revealed that women with stillbirth were less likely to report having received a postnatal check [54]. The difference in these findings, if true, may indicate a disparity in the perception of postpartum care received by women by both health care providers and the women themselves.

However, even when all these health care recommendations are taken to be protective by World Health Organization in its guidelines [6–8], some of these have not been statistically significant in this study. Taking an ultrasound scan; having blood pressure, weight and pallor assessment; and receiving iron/folic acid, fansidar, and tetanus toxoid during pregnancy have not been observed to be statistically associated with adverse outcomes in this study. Therefore, this study may not be powered enough to explain their influence on perinatal death.

Furthermore, the study has not observed an association between perinatal death and perinatal care except for fetal status monitoring during pregnancy. This may have resulted from the low occurrence of perinatal deaths shown in this paper. This study may also not be powered enough to explain perinatal care influence on perinatal death. Further studies with higher statistical power could re-assess the association between perinatal care and perinatal death.

This study had some limitations. First, this study extracted the data on care from patient records and was not corroborated by patient or provider interviews. It is possible that information recorded did not exactly reflect the care provided to mothers during these periods of care. In addition, there is no data on direct observation of the care processes which would have explained or validated the data extracted from the patient records. This could have affected the assessment of the relationships between care processes and birth outcomes. Secondly, the source of data also makes it hard to appreciate the in-depth factors that could influence occurrence of perinatal deaths including personal factors like beliefs, values, experiences, and ability to access services. Thirdly, the study did not include women who were referred for childbirth from other facilities and did not attend antenatal care in the study hospitals yet perinatal deaths are common among these women [29, 30]. Follow up on baby status after birth was also for 24 h only instead of 7 days. Therefore, the estimated perinatal death rates may be lower than the actual values in this setting. The study did not capture information on type of pregnancy, existing co-morbidities, gestation age at birth, care given to the newborn, and the effect of postnatal care to the mother on the newborn which could have influenced the occurrence of perinatal deaths among mothers. Lastly, it was a cross sectional study that merely provided a snapshot of the care processes and their associated birth outcomes in these facilities. To mitigate the limitations, interviews were held with women and health care providers to validate the care provided and observations of care provided during the process of data extraction was done to give insight on the context in which care was provided, although this was limited and not documented and thus not included in the study findings. Despite the limitations, results from the study are still valid as they highlight the gaps in care, the linkage between care provided and outcome, and make suggestions for strengthening perinatal care, which provides a basis for further studies. To our knowledge, this could be the first study to document association between perinatal care recommendations and perinatal death in a resource limited setting.

## Conclusion

The study shows a high perinatal mortality rate in Bunyoro although lower than the national average. Fetal status monitoring during the antenatal period significantly reduces occurrence of perinatal deaths. Having many antenatal contacts and blood tested for HIV, hemoglobin, and syphilis during antenatal also reduces the likelihood of having perinatal deaths even when not statistically significant. Generally, monitoring women during labour and childbirth for fetal condition, maternal condition, and labour progress reduces their chance of experiencing perinatal death despite not being statistically significant. Therefore, to address perinatal mortality in this region of Uganda, strategies should focus on the quality of care provided to women by health care providers, particularly care that shows the status of the fetus and the woman during pregnancy and childbirth. Efforts should also be made to empower women so as to enhance their ability to seek for perinatal services.

### Electronic supplementary material

Below is the link to the electronic supplementary material.

Supplementary Material 1

Supplementary Material 2

## Data Availability

The datasets generated or analyzed during this study are included in this published article (and its supplementary information files).

## Abbreviations

ANC: Antenatal care
BBA: Birth before arrival
BP: Blood Pressure
FHR: Fetal Heart Rate
FH: Fundal Height
Fe/Fo: Iron/Folic acid
PR: Pulse Rate
SP: Sulphadoxine Pyrimethamine (Fansidar)
Wt: Weight
UGX: Uganda Shillings
