# Supplementary material for: Perinatal care and its association with perinatal death among women attending care in three district hospitals of western Uganda
Source: BMC Pregnancy Childbirth. 2024 Feb 6;24:113. doi: 10.1186/s12884-024-06305-5 (PMC10845583; doi:10.1186/s12884-024-06305-5)
Supplement: Supplementary file 1 — Supplementary Material 1 [file 12884_2024_6305_MOESM1_ESM.pdf]

Supplement Table 1. Perinatal Care received by Women stratified by outcomes

**Care components**	Live birth<br>N=853<br>n (%)	Perinatal death<br>N=19<br>n (%)	Total<br>N=872<br>n (%)
**Antenatal care**			
ANC Contacts			
< 8 contacts	844(98.9)	19(100.0)	863(99.0)
8 or more contacts	9(1.1)	0(0.0)	9(1.0)
ANC Contacts			
< 4 contacts	397(46.5)	9(47.4)	406(46.6)
4 or more contacts	456(53.5)	10(52.6)	456(53.5)
ANC initiation			
1st trimester	70(8.2)	3(15.8)	73(8.4)
After 1st trimester	783(91.8)	16(84.2)	799(91.6)
ANC Prophylaxis (Iron/Folic, Fansidar, TT)			
No	129(15.1)	2(10.5)	131(15.0)
Yes	724(84.9)	17(89.5)	741(85.0)
Blood tests (HIV, Syphilis, Hb)			
No	801(93.9)	18(98.7)	819(93.9)
Yes	52(6.1)	1(5.3)	53(6.1)
Urine test (Glucose, Proteins)			
No	826(96.8)	19(100.0)	845(96.9)
Yes	27(3.2)	0(0.0)	27(3.1)
Ultrasound scan			
No	598(70.1)	12(63.2)	610(70.0)
Yes	255(29.9)	7(36.8)	262(30.0)
Fetal status monitoring (FH, FHR, Lie, Position)			
No	18(2.1)	2(10.5)	20(2.3)
Yes	835(97.9)	17(89.5)	852(97.7)
Maternal status monitoring (BP, Weight, Pallor)			
No	736(86.3)	14(73.7)	750(86.0)
Yes	117(13.7)	5(26.3)	122(14.0)
**Intrapartum care**			
Fetal condition assessment (FHR)			
No	204(23.9)	8(42.1)	212(24.3)
Yes	649(76.1)	11(57.9)	660(75.7)
Maternal condition assessment (BP, PR)			
No	354(41.5)	13(68.4)	367(42.1)
Yes	499(58.5)	6(31.6)	505(57.9)
Labour progress assessment (cervical dilatation, descent of presenting part, uterine contraction)			
No	252(29.5)	9(47.4)	261(29.9)
Yes	601(70.5)	10(52.6)	611(70.1)
**Postpartum care**			

Uterine assessment (uterine contraction, vaginal bleeding, FH)			
No	794(93.1)	15(79.0)	809(92.8)
Yes	59(6.9)	4(21.0)	63(7.2)
Maternal status assessment (BP, PR, Temperature)			
No	332(38.9)	7(36.8)	339(38.9)
Yes	521(61.1)	12(63.2)	533(61.1)
